# The cost-effectiveness of exercise referral schemes

**DOI:** 10.1186/1471-2458-11-954

**Published:** 2011-12-26

**Authors:** Nana K Anokye, Paul Trueman, Colin Green, Toby G Pavey, Melvyn Hillsdon, Rod S Taylor

**Affiliations:** 1 Health Economics Research Group (HERG), Brunel University, Uxbridge, Middlesex UB8 3PH, UK; 2 Peninsula College of Medicine and Dentistry, University of Exeter, Veysey Building, Salmon Pool Lane, Exeter EX2 4SG, UK; 3 School of Sport and Health Sciences, University of Exeter, St Luke's, Exeter EX1 2 LU, UK

## Abstract

**Background:**

Exercise referral schemes (ERS) aim to identify inactive adults in the primary care setting. The primary care professional refers the patient to a third party service, with this service taking responsibility for prescribing and monitoring an exercise programme tailored to the needs of the patient. This paper examines the cost-effectiveness of ERS in promoting physical activity compared with usual care in primary care setting.

**Methods:**

A decision analytic model was developed to estimate the cost-effectiveness of ERS from a UK NHS perspective. The costs and outcomes of ERS were modelled over the patient's lifetime. Data were derived from a systematic review of the literature on the clinical and cost-effectiveness of ERS, and on parameter inputs in the modelling framework. Outcomes were expressed as incremental cost per quality-adjusted life-year (QALY). Deterministic and probabilistic sensitivity analyses investigated the impact of varying ERS cost and effectiveness assumptions. Sub-group analyses explored the cost-effectiveness of ERS in sedentary people with an underlying condition.

**Results:**

Compared with usual care, the mean incremental lifetime cost per patient for ERS was £169 and the mean incremental QALY was 0.008, generating a base-case incremental cost-effectiveness ratio (ICER) for ERS at £20,876 per QALY in sedentary individuals without a diagnosed medical condition. There was a 51% probability that ERS was cost-effective at £20,000 per QALY and 88% probability that ERS was cost-effective at £30,000 per QALY. In sub-group analyses, cost per QALY for ERS in sedentary obese individuals was £14,618, and in sedentary hypertensives and sedentary individuals with depression the estimated cost per QALY was £12,834 and £8,414 respectively. Incremental lifetime costs and benefits associated with ERS were small, reflecting the preventative public health context of the intervention, with this resulting in estimates of cost-effectiveness that are sensitive to variations in the relative risk of becoming physically active and cost of ERS.

**Conclusions:**

ERS is associated with modest increase in lifetime costs and benefits. The cost-effectiveness of ERS is highly sensitive to small changes in the effectiveness and cost of ERS and is subject to some significant uncertainty mainly due to limitations in the clinical effectiveness evidence base.

## Background

Insufficient physical activity is an important public health issue in England as it is associated with an increased risk of developing over 20 health conditions including coronary heart disease (CHD), cancer, diabetes, and stroke [1-4] and is rated among the top ten leading causes of death in high-income countries [5]. In England, physical inactivity is estimated to cost the economy around 8.3 billion pounds annually, of which between 1 and 1.8 billion pounds is associated with the treatment of physical inactivity related diseases [6]. In spite of the negative impacts of physical inactivity, only 39% of men and 29% of women in England reported meeting the recommended level to be considered 'physically active', as defined by guidance from the Chief Medical Officer, whilst based on accelerometer data, only 6% of men and 4% of women met the recommended level [7].

Over the past decade, exercise referral schemes (ERS) have become one of the most common interventions used to promote physical activity in primary care [8,9]. In an ERS, people who are sedentary and/or have risk factor(s) for conditions known to benefit from physical activity (e.g. high blood pressure) are referred by a primary care professional to a third party service (often a sports centre or leisure facility), which then prescribes and monitors an exercise programme tailored to the individual needs of the patients [9].

To date, there is limited evidence on the cost-effectiveness of ERS. A systematic review identified four previous economic evaluations [10]. These comprised three trial-based economic evaluations of ERS [11-13] and one model-based evaluation [8] of the cost-effectiveness of brief interventions in primary care to promote physical activity, including ERS. Whilst the evidence base suggests that exercise referral is a cost-effective intervention in sedentary but otherwise healthy populations there are a number of shortcomings associated with the evidence. First, as the authors of each of the studies acknowledge, there is significant uncertainty around estimates of cost-effectiveness, mainly due to limitations in the effectiveness evidence. Second, the evidence tends to focus on sedentary but otherwise healthy individuals, while a number of individuals are currently referred to an ERS with a diagnosed condition, such as coronary heart disease or depression [14].

This paper aims to examine the cost-effectiveness of ERS in promoting physical activity compared to usual care in a primary care setting. Our analysis uses previous research as a point of departure, and builds on this through use of evidence synthesis and through further analysis of the cost-effectiveness of ERS in individuals with pre-existing conditions, which is intended to reflect the use of ERS in practice in the UK.

## Methods

### Modelling approach

**Figure 1:**
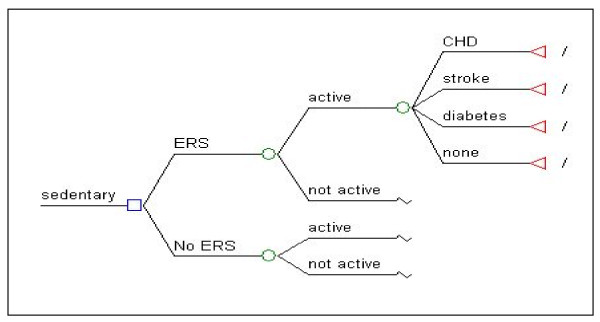
**Diagram of model structure**.

A decision analytic model was developed to examine the cost effectiveness of ERS. The model considers a cohort of individuals who are exposed to ERS compared to a control group with no ERS. The modelling framework estimates the likelihood of becoming physically active and examines the effects of physical activity/inactivity on the development of conditions which are known to be associated with level of physical activity. Specifically, the model considers the impact of ERS on the development of coronary heart disease, stroke and type II diabetes. Whilst many other conditions are thought to be associated with physical activity, these three conditions were selected on the basis that there is robust quantifiable evidence on the relationship between physical activity and their incidence [15]. Figure 1 illustrates the model structure, which is a based on a previously developed policy-relevant cost-effectiveness model [8]. This structure was reviewed against best practice principles for economic modelling and considered suitable [10].

### Population

The model considers a cohort of individuals, aged between 40-60 years, who present in a sedentary state. The age of the population was selected to reflect the evidence on the effectiveness of ERS [10].

### Perspective and time horizon

The model adopts an NHS and personal social services perspective (third-party payer perspective), as used by the National Institute for Health and Clinical Excellence (NICE) in it's reference case for cost-effectiveness analysis in health technology appraisal [2]. A lifetime time horizon is adopted to capture future costs and to acknowledge the benefits of physical activity. Future costs and benefits are discounted at a rate of 3.5% per annum [2].

### Intervention and comparator

ERS was assumed to take the form of a structured programme of exercise based in a leisure centre incorporating monitoring of individual performance, as is mainly the case in current practice [10]. Individuals are assumed to have been referred to the scheme by a primary care professional. The comparator was usual care in a primary care setting. It should be acknowledged that individuals who are not exposed to ERS may choose to become physically active.

### Effectiveness of ERS/comparator

Evidence of the effectiveness of ERS, compared with usual care, measured in terms of the probability of moving from a sedentary state to an active state, was derived from the meta-analysis conducted as part of a recent review of the effectiveness literature for ERS [10]. This was based on 'intention-to-treat' analyses, which adjusted for attrition, and showed ERS to be associated with a higher probability (relative risk (RR): 1.11; 95% CI: 0.99, 1.25) of being active compared with usual care. The active state is defined in line with the effectiveness literature and physical activity for health guidance [16] i.e. doing at least 90-150 min of at least moderate intensity physical activity per week. Thus, a sedentary lifestyle corresponds not only to non-participation in physical activity but also to participation below the requisite amount. The active state is assumed to last long enough to enable health benefits to be obtained, although this remains undefined given the inadequate evidence on the dose response relationship between the number of years being physically active and the incidence of long-term outcomes. Previous analyses of behaviour change have referred to this scenario as 'fully engaged' [17] to describe an individual who makes lasting changes to their lifestyle following an intervention.

### Risks of developing health states associated with inactivity

Evidence of the effect of physical activity on the development of the outcomes considered in the model (CHD, stroke and type II diabetes) is derived from a systematic review [8] and from the Health Survey for England (HSE) 2006 (survey year focused on cardiovascular disease and risk factors). HSE is the main data source on morbidities in England [18]. The probability of developing CHD, stroke or type II diabetes among sedentary individuals is generated from the prevalence of these conditions in that population, using the HSE 2006 data to inform these probabilities (Table 1). The probability of developing the health states among active individuals are derived using RR estimates identified from literature review [8,19,20] and subsequent adjustment (dividing the probabilities for the sedentary population by the RR from the literature) of the probability for each condition in the ERS cohort. The physical activity levels and study population used to measure the RR estimates match those of the cohort under consideration in this study.

**Table 1:** Estimates of the inputs to the model

Input	Value	Data source
***Probability of experiencing an outcome associated with physical activity***		

Probability of experiencing CHD when active	0.014	HSE [21]; Shaper et al. [19]

Probability of experiencing CHD when sedentary	0.027	HSE [21]; Shaper et al. [19]

Probability of experiencing stroke when active	0.011	HSE [21]; Herman et al. [20]

Probability of experiencing stroke when sedentary	0.015	HSE [21]; Herman et al. [20]

Probability of experiencing type II diabetes when active	0.022	HSE [21]; NICE [8]

Probability of experiencing type II diabetes when sedentary	0.044	HSE [21]; NICE [8]

***Inputs used in calculating QALYs/treatment costs***		

Utility/health state value of being in CHD state	0.55	Kind et al. [22]; NICE [8]

Utility/health state value of being in stroke state	0.52	Kind et al. [22]; NICE [8]

Utility/health state value of being in type II diabetes state	0.7	Kind et al. [22]; NICE [8]

Utility/health state value of being in a non-disease health state	0.83	Kind et al. [22]; NICE [8]

Average age of cohort (in years)	50	HSE [18]

Average age of mortality (in years)	84	ONS [23]

Assumed average age of onset of a disease health state (in years)	55	NICE [8]

Life years remaining after onset of CHD	18.41	NICE [8]; ONS [23]

Life years remaining after onset of stroke	5.12	NICE [8]; ONS [23]

Life years remaining after onset of type II diabetes	28.13	NICE [8]; ONS [23]

Lifetime treatment costs*/QALYs associated with health states (per person)		

Lifetime treatment costs associated with CHD state	£17,728	NICE [8]

Lifetime treatment costs associated with stroke state	£1,965	DH [22]

Lifetime treatment costs associated with type II diabetes state	£50,309	Currie et al. [21]

Lifetime treatment costs associated with non-disease health state	-	-

QALYs associated with CHD state	9.94	Kind et al. [23]; NICE [8]

QALYs associated with stroke state	5.15	Kind et al. [23]; NICE [8]

QALYs associated with type II diabetes state	14.18	Kind et al. [23]; NICE [8]

QALYs associated with non-disease health state	17.18	Kind et al. [23]; NICE [8]

*Costs are in 2010 prices.

### ERS/intervention costs

The cost of the ERS intervention was derived from previously published research identified as part of a recently published systematic review [10] which identified a detailed micro-level costing exercise for a leisure centre based ERS [12]. Isaacs et al. [12] reported resource use in a health service/local authority that consists of provision of facilities, exercise trainers and administrative support. Cost estimates are up-rated to 2010 prices, using the consumer price index, for the current analyses. The validity of the resource use and cost estimates employed for ERS were assessed by an expert advisory group (including clinicians, exercise scientists and health economists) and judged to be representative of ERS schemes in current practice. No attempt was made to estimate a net cost of the intervention which subtracts any cost savings that might result from ERS from the cost of the intervention. When this was explored in Isaacs et al. [12] and Gusi et al. [11], there was no clear evidence of a change in health care utilisation (e.g. medications, hospital or primary care) as result of the intervention.

### Treatment costs with CHD, stroke and type II diabetes

Total lifetime treatment costs were estimated using published cost estimates (identified through a systematic review) for the annual cost associated with CHD, diabetes, and stroke [8,21,22], and assumptions on age of onset and life-expectancy combined with estimates of the annual cost of treating an individual with the condition [15]. It was assumed that the treatment cost of stroke, unlike the other health states was an event cost because the direct costs associated with treatment of stroke tend to occur once, rather than a recurring cost. This is acknowledged as a simplification in the model, as in reality there are likely to be acute and ongoing costs associated with stroke.

### Primary outcome measure (QALY)

The primary outcome of the economic evaluation is expressed in terms of the incremental cost per quality-adjusted life-year (QALY). Estimates of the QALYs associated with each of the conditions in the model are derived using health state values for each condition [8,23] and data on life-expectancy after onset of the condition [24]. Life-expectancy is derived by applying data on average age of onset for each condition (Table 1).

### Assessment of uncertainty

Uncertainty in parameter estimates was explored through the use of deterministic and probability sensitivity analyses. The deterministic sensitivity analysis included one-way, scenario and extreme values analysis. In addition, uncertainties around parameters considered to be key drivers of the cost-effectiveness of ERS were addressed simultaneously using probabilistic sensitivity analyses (PSA). The parameters that had different unit values in the 2 arms of the model (i.e. probability to be active and probability to get the disease conditions) were specified as incremental differences between the 2 arms and not absolute values. The intuition is that the distributions of these parameters may be correlated and hence representing them as absolute values may overestimate the uncertainty. The distributions and their respective calculation of alpha and beta calculations were based on [25]. In cases where there were no data on standard errors the standard approach of using 10% of mean estimates as standard error was followed [26] The data adopted in the probabilistic analysis are reported in Table 2.

**Table 2:** Probabilistic sensitivity analysis inputs

Parameters	Deterministic	Standard error	Distribution	Alpha	Beta
Incremental probability to be active	0.048	0.0048	beta	95.152	1887.181

Incremental probability to experience CHD	0.013	0.0013	beta	98.687	7492.621

Incremental probability to experience stroke	0.004	0.0004	beta	99.596	24799.4

Incremental probability to experience diabetes	0.022	0.0022	beta	97.778	4346.677

Treatment discounted cost of CHD	17728.031	1772.803	gamma	100	177.280

Treatment discounted cost of stroke	1965.165	196.517	gamma	100	19.652

Treatment discounted cost of diabetes	50309.426	5030.943	gamma	100	503.094

Discounted QALY for CHD health state	9.942	0.994	gamma	100	0.099

Discounted QALY for stroke health state	5.148	0.515	gamma	100	0.051

Discounted QALY for type II diabetes health state	14.182	1.418	gamma	100	0.142

Cost of intervention	222	37.9	gamma	34.311	6.470

Source: Briggs et al (25)

### Subgroup analyses in individuals with pre-existing conditions

Sub-group analyses included an assessment of the cost effectiveness of ERS in sedentary 40-60 year olds individuals with a diagnosed condition known to benefit from physical activity. Obesity, hypertension and depression were identified as the three most common conditions reported with participation in ERS [14] and were included in the analysis.

### Model validation

Two main procedures involving internal validation and peer review were employed to check the validity of the model [27]. The former consisted of simulating a series of changes in the input values that are likely to vary the results of the model with checks to see that the impacts on the results are expected. For example, setting all QALY parameters to zero, and checking if the output of the QALYs in each arm is zero. In addition to this, the model was replicated and compared using TreeAge and excel software, and subject to a process of internal peer review, including consistency checks, across the research team. The validation process included peer review by a modeller, unrelated to the research team, who understood the complexities of the model and who was able to scrutinise the spreadsheet of the model and the formulae behind it.

## Results

### Estimates of effectiveness/costs of ERS

Table 3 summarises the estimates of the effectiveness of ERS on physical activity levels and overall intervention costs associated with ERS.

**Table 3:** Estimates of effectiveness and intervention costs of ERS

Inputs	Value	Data source
***Effectiveness***		

Probability of becoming active after exposure to ERS	0.345	Pavey et al. [10]

Probability of becoming active after exposure to usual care	0.297	Pavey et al. [10]

***Intervention costs***		

Cost of the intervention per participant to the providers	£222^a^	Pavey et al. [10]

^a^In 2010 prices (estimates used in model)

### Estimates of the outcomes associated with physical activity

Table 1 reports the derivation of the outcomes associated with physical activity. This includes the probability of experiencing an outcome (CHD, stroke or type II diabetes), utility values, and life years associated with each outcome.

The estimates of lifetime treatment costs and QALYs for an individual in each health state are summarised in Table 1. Among the conditions included in the model, type II diabetes incurred the largest treatment cost and stroke the least, although it should be noted that stroke was considered as a one off clinical event whilst other chronic outcomes were associated with ongoing treatment costs.

### Estimating the cost-effectiveness of ERS

Table 4 shows the estimated incremental cost-effectiveness ratio (ICER) of the base-case analysis, using a cohort of 1,000 individuals and a lifetime horizon. Total costs and outcomes are divided by the cohort size (1,000) to generate per person estimates of costs and benefits. The ICER was calculated with respect to the standard comparator 'usual care'. Compared with usual care, ERS is more expensive as it incurs additional mean lifetime costs of £170 per person, but is more effective leading to a lifetime mean QALY gain of 0.008 per person. The mean cost per QALY of ERS compared with usual care is £20,876.

**Table 4:** Base-case cost-effectiveness results comparing ERS with usual care

	ERS	Usual care	Difference	Incremental cost per QALY (ICER)
Lifetime total healthcare costs per person^a^	£2,492	£2,322	£170	£20,876
	
Total QALYs per person	16.743	16.735	0.008	

^a^In 2010 prices

### Deterministic sensitivity analysis

Table 5 shows the impact of the variation in parameter estimates (one-way analysis) on the cost-effectiveness of ERS. Assuming a less intensive ERS or more effective ERS resulted in an ICER below £30,000 per QALY and lower than the base-case. On the other hand, including intervention costs to participants led to an ICER above £30,000 per QALY, whilst a less effective ERS resulted in ERS being dominated by usual care (negative ICER)-that is ERS is more expensive and leads to loss of health gains. The findings of the scenario analysis are presented in Table 5. In the worst case scenario, ERS was dominated by the comparator. In the best case scenario, the ICER fell to under £700 per QALY.

**Table 5:** Cost-effectiveness results (after deterministic sensitivity analyses) comparing ERS with usual care

Parameters/scenarios	How data was adjusted for in the model	Incremental cost per person	Incremental effect per person (QALY)	ICER
Base case analysis	-	£170	0.008	£20,876

Parameters				

Intervention costs to participants	Costs of intervention was varied from £222 to £342 (including costs to providers and participants)	£290	0.008	£35,652

Less intensive ERS	Costs of intervention was varied from £222 to £110	£58	0.008	£7,085

Effectiveness of ERS (based on lower limit of 95% CI)	Probability of becoming active after exposure to ERS was varied from 0.336 to 0.294	£226	-0.001	Dominated*

Effectiveness of ERS (based upper limit of 95% CI)	Probability of becoming active after exposure to ERS was varied from 0.336 to 0.371	£122	0.015	£7,947

Scenarios				

Worst cases of cost and effectiveness	Worst case cost (£342) and worst case effectiveness (0.294)	£346	-0.001	Dominated*

Best cases of cost and effectiveness	Best case cost (£110) and best case effectiveness (0.371)	£10	0.015	£679

Worst case cost and best case effectiveness	Best case cost (£110) and worst case effectiveness (0.294)	£242	0.015	£15,734

Best case cost and worst case effectiveness	Worst case cost (£342) and best case effectiveness (0.371)	£114	-0.001	Dominated*

*ERS more costly and less effective than control

### Probabilistic sensitivity analysis

**Figure 2:**
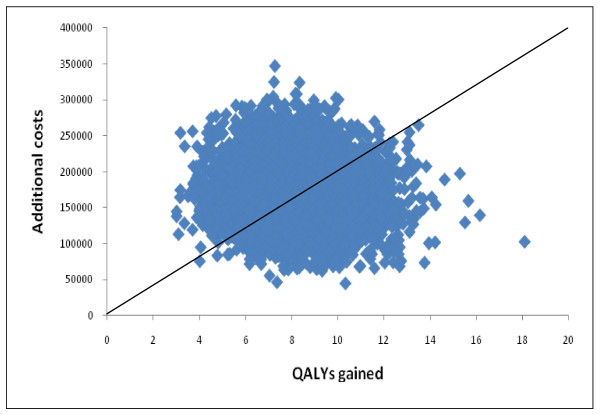
**Cost-effectiveness plane**.

A scatter plot of the probabilistic data, showing simulated estimates of cost difference against QALY difference between ERS and usual care, is provided in Figure 2. The scatter plot shows that all the simulations generated an improved effectiveness of ERS but also at higher cost (i.e. all points were in the north-east quadrant of the cost-effectiveness plane). This reflects the relatively modest uncertainty around the cost of the intervention and assumptions about the distribution of uncertainty around the estimates of effect size.

**Figure 3:**
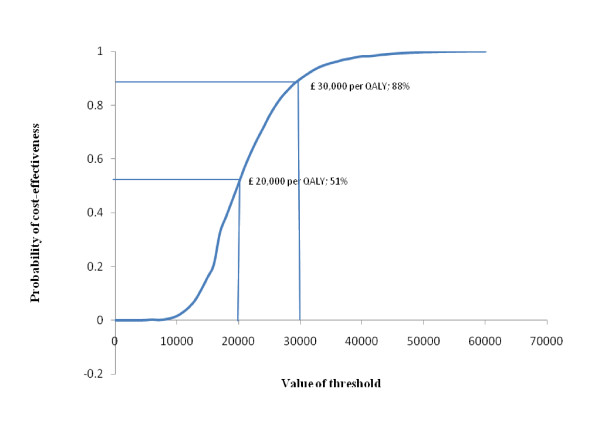
**Cost-effectiveness acceptability curve showing the probability of cost-effectiveness for ERS at varying levels of threshold**.

A judgment on the cost-effectiveness of ERS, from a decision-maker context, will depend on the maximum amount decision makers are willing to spend to obtain an additional unit of effectiveness (in this case, a QALY). This judgement can be informed through the presentation of a cost-effectiveness acceptability curve, as presented in Figure 3. At a threshold of £20,000 per QALY, there is a 0.508 probability that ERS is cost-effective. This increases to 0.879 when a threshold of £30,000 per QALY is considered.

### Subgroup analysis in individuals with pre-existing conditions

Table 6 shows the probabilities of experiencing the health states in the disease-specific cohorts. For each of the conditions considered, the estimated ICER is lower than the base case, reflecting the increased likelihood of developing one of the morbidities considered in the model if the individual has a pre-existing condition (Table 7). Compared with usual care, ERS in these cohorts remains more costly (albeit less so than in a general population cohort). In terms of effectiveness, ERS (compared with usual care) is more effective leading to improved QALY gains which are higher than in the base case (ranging from 0.011 to 0.017). The cost per QALY of ERS compared with usual care is between £8,414 and £14,618 and thus can be considered cost-effective at the £20,000 per QALY threshold.

**Table 6:** Inputs used in the subgroup analysis model

Cohort	Inputs	Value	Data source
Obese	Probability of experiencing CHD when active	0.0259	HSE [28]; Hu et al. [18]
	
	Probability of experiencing CHD when sedentary	0.0376	HSE [28]; Hu et al. [18]
	
	Probability of experiencing stroke when active	0.0259	HSE [28]; Hu et al. [18]
	
	Probability of experiencing stroke when sedentary	0.0376	HSE [28]; Hu et al. [18]
	
	Probability of experiencing type II diabetes when active	0.0756	HSE [28]; Hu et al. [18]
	
	Probability of experiencing type II diabetes when sedentary	0.0986	HSE [28]; Hu et al. [18]

Hypertensive	Probability of experiencing CHD when active	0.060	HSE [28]; Hu et al. [29]
	
	Probability of experiencing CHD when sedentary	0.074	HSE [28]; Hu et al. [29]
	
	Probability of experiencing stroke when active	0.060	HSE [28]; Hu et al. [29]
	
	Probability of experiencing stroke when sedentary	0.074	HSE [28]; Hu et al. [29]

Depressive	Probability of experiencing CHD when active	0.0336	HSE [28]; Surtees et al. [30]
	
	Probability of experiencing CHD when sedentary	0.0801	HSE [28]; Surtees et al. [30]

**Table 7:** Cost-effectiveness results (disease specific cohorts) comparing ERS with usual care

Cohort	Incremental cost per person(£)	Incremental effect per person(QALY)	ICER (£)
Obese	£168	0.011	£14,618

Hypertensive	£168	0.013	£12,834

Depressive	£147	0.017	£8,414

## Discussion

Our analysis estimates the cost-effectiveness of ERS using a cost utility analysis framework. Our base case assumptions result in a favourable cost-effectiveness ratio of £20,876 per QALY gained from ERS compared to usual care. The typical cost effectiveness threshold for UK ranges from £20,000 to £30,000 per QALY. However, ICERs were highly sensitive to plausible variations in the relative risk for change in physical activity and cost of ERS. The cost-effectiveness of ERS appears to improve when targeted at individuals with a pre-existing condition known to benefit from increased physical activity (i.e. £14,618/QALY in sedentary obese individuals, £12,834/QALY in sedentary hypertensives and £8,414/QALY for sedentary individuals with depression). This suggests that it might be possible to target ERS to individuals with pre-existing conditions in whom the payoffs/impact may be higher. However, there remain some major uncertainties over whether the evidence used to populate the model, derived from the meta-analysis, is applicable to these groups. There may be good reason to believe that uptake, adherence and effectiveness might differ according to the characteristics of the recipients. We have attempted to adjust the model to take into account differences in the rate of long-term illnesses, but no data were identified as part of the effectiveness review to allow for adjustment of the effect of ERS in different populations. There is a pressing need for better primary evidence to inform these uncertainties.

Whilst our cost-effectiveness estimates suggest that ERS is a cost effective use of National Health Service (NHS) resources, it should be noted that the individual level lifetime QALY gains are relatively modest (less than 0.01 in our base case analysis). This estimate is predicated on the evidence of effectiveness derived from a meta-analysis [10], which has provided the most robust estimate to date of the effectiveness of ERS compared to usual care. However, it should be acknowledged that the cost-effectiveness analysis is attempting to capture lifetime benefits based on evidence of relatively modest effect sizes derived from short-term studies. Any such analysis inevitably involves some assumptions about the degree to which behaviour change is lasting and fails to consider other health behaviours which may impact on long-term outcomes. The result is that the cost-effectiveness analysis estimates that ERS has a modest lifetime cost and a marginal lifetime QALY gain. Even small changes in the source data used to populate the model, particularly evidence of effect size and cost, may lead to significant changes in the resulting ICER. This can best be illustrated through consideration of the net benefit calculation. If we value each QALY gained at £30,000 and accept that our analysis is generating a lifetime QALY gain of approximately 0.008 in most cases, then the value of the benefits generated in monetary terms is approximately £240 which exceeds the cost of the intervention. However, even a modest change in the lifetime QALY gain, to 0.007 would result in the costs exceeding the benefits making the cost-effectiveness of ERS questionable.

There are a number of limitations in the analysis that need to be acknowledged. In some respects the analysis can be considered to be conservative as it includes only a small number of conditions which are associated with physical activity. The inclusion of other conditions, such as musculoskeletal disease and mental health, are expected to further improve the cost-effectiveness of ERS. These conditions are excluded from the current analysis due to limitations in the available data on the relationship between their incidence and physical activity. Additional developments of this model to adopt a wider perspective via the incorporation of 'non-health' outcomes associated with ERS slightly further improved the cost-effectiveness of ERS to a base case ICER of £17,032/QALY (see Pavey et al. [10] for detail). In the analyses considering the 'non-health' outcomes, impacts of PA were captured as: (a) reduced absenteeism at work and disbenefits such as injuries and disability, and (b) process utility directly attributable to increased exercise. The former set of outcomes were obtained through synthesis of the literature to identify estimates of the magnitude of their associations with physical activity [10] and accounted via a descriptive cost consequences analysis. The process utility was included as a one-off 'feel good' benefit (QALY gain) associated with being physically active, and was estimated via regression analyses using HSE 2008 data related to EQ-5D and self-reported physical activity, with uncertainty in this estimate tested via sensitivity analyses. Conversely, there are a number of assumptions which could be considered to be favourable to ERS, notably, the assumption relating to the lasting effect of physical activity.

Sensitivity analyses provide some reassurance that the net effect of these assumptions is modest and that the incremental cost-effectiveness of ERS remains below £30,000 per QALY under most scenarios. Furthermore, our findings are largely consistent with previous analyses of ERS which have suggested that ERS results in modest increases in QALYs (via adverse health events avoided) at a relatively low cost. Previous studies have tended to conclude that ERS is a cost-effective use of resources, although they too have highlighted uncertainty in evidence based and the analytical framework used. Isaacs et al. [12] presented results in the form of an incremental cost per unit change in SF-36 score, with the authors concluding that in comparison with controls, ERS led to an incremental cost of £19,500 per unit change in SF-36 score at 6 month follow-up. Given the outcome measure adopted in the study it is not possible to make helpful comparison with our own findings, although it should be noted that this study also found only a modest change in health status. In contrast, the study by Gusi et al. [11] showed that ERS resulted in an incremental QALY gain of 0.132 over a 6 month period as measured by change in the EQ-5D, at an incremental cost of €41 per participant, generating an ICER of €311 per QALY. The individuals in this study were obese and/or depressed and the findings may provide further evidence to suggest that physical activity can have process benefits, i.e. health status gains (independent of other preventative effects) far greater than those suggested by our own analysis. However, no attempt was made to ascertain whether the benefits might be sustained beyond the study period. The findings presented by NICE [8] showed ERS compared with controls led to an incremental cost per person of £25.10 and a lifetime QALY gain of 0.31 per person equating to an incremental cost per QALY of £81. We are inclined to relate our findings more directly to the NICE [8] analysis because of similarities in the methods used in both studies. For example, the model used in our study was based on the model used by NICE [8]. The analysis conducted for NICE showed a greater QALY gain than our own findings. This might be partially explained by the inclusion of colon cancer as an additional outcome in the NICE model. In addition to this, the NICE model adopted higher estimates of the effectiveness of ERS than our analysis (RR of becoming active of 1.60 vs 1.11 herein) and there are differences in the handling of uptake and adherence between the two analyses. Coupled with a lower estimated cost of ERS this result in the NICE analysis generating improved ICERs compared to our own findings. In testing our own model we sought to reproduce the findings of the NICE model by incorporating the improved effectiveness of ERS. Despite slight differences in the modelling approach it produced relatively consistent findings. Whilst we have based our approach to modelling the cost-effectiveness of ERS on the model structure used by NICE, we believe that the meta-analysis of effectiveness used in the current economic analysis has resulted in more robust input data and ultimately more accurate estimates of the cost-effectiveness of ERS [10].

However, all of the studies reported above suggest that ERS is associated with only small mean differences in lifetime costs and benefits, giving rise to the resulting ICER being very sensitive to small changes in the relative risk of becoming physically active, together with small changes in other data inputs. This highlights the main limitation of this research, namely the limited evidence to show that ERS has a significant and lasting effect on participation in physical activity. Related to this, the model assumed that the active state last long enough to enable health benefits to be obtained and this could not be addressed in the sensitivity analysis due lack of data and the type of model used. Decision analytic models may not be well suited to interventions which involve complex behaviour change components. Individual level simulation models which can detect changes in individual behaviours over time may better address cost effectiveness. However, there will always be a trade-off between developing a simple model, which can be populated and acknowledges its limitations versus a more complex model which may be a better representation of reality but can only be partially populated and may result in greater uncertainty. The fundamental issue which needs to be addressed is improvements in the source data on the effectiveness of ERS.

Further research is urgently required to examine the effectiveness of ERS with a particular focus on 1) how to motivate individuals to participate in ERS; 2) identify sub-groups of the sedentary population who are most able to benefit from ERS; 3) identify factors that are likely to lead to sustained increased in physical activity and changes in lifestyle. In the absence of robust evidence on these, the economic case for ERS remains encouraging but ultimately equivocal.

## Conclusions

This study examines the cost-effectiveness of ERS in promoting physical activity compared to usual care in a primary care setting. Using a cost utility analysis framework, the study uses previous research as a point of departure, and builds on this through use of evidence synthesis and through further analysis of the cost-effectiveness of ERS in individuals with pre-existing conditions, which is intended to reflect the use of ERS in practice in the UK. ERS is associated with modest increase in lifetime costs and benefits. Compared to usual care, the base-case ICER for ERS was £20,876/QALY in sedentary individuals with at least one lifestyle risk factor and £14,618/QALY in sedentary obese individuals, £12,834/QALY in sedentary hypertensives and £8,414/QALY for sedentary individuals with depression. However, cost-effectiveness of ERS is highly sensitive to small changes in the effectiveness and cost of ERS and is subject to some significant uncertainty mainly due to limitations in the clinical effectiveness evidence base. Therefore, further research on the clinical effectiveness of ERS is strongly recommended.

## Competing interests

The authors declare that they have no competing interests.

## Authors' contributions

NKA and PT undertook the economic modelling with support from CG, drafted the first manuscript and coordinated its revision. TP and RT directed the project, analysed the data for the clinical effectiveness, helped identify data sources and assisted in drafting and revising the manuscript. MH provided an advisory role and assisted in drafting and revising the manuscript. All authors read and approved the final manuscript.

## Pre-publication history

The pre-publication history for this paper can be accessed here:

http://www.biomedcentral.com/1471-2458/11/954/prepub
